# Supplementary material for: Michigan men’s diabetes project II: Protocol for peer-led diabetes self-management education and long-term support in Black men
Source: PLoS One. 2023 Mar 2;18(3):e0277733. doi: 10.1371/journal.pone.0277733 (PMC9980828; doi:10.1371/journal.pone.0277733)
Supplement: S1 File — (DOCX) [file pone.0277733.s002.docx]

**Michigan Men’s DIabetes Project 2 P IRB Protocol**

**1.0 Phase I, Phase II, and Phase III Research Plans**

**Phase I (Development)**: HUM00200496.

**Phase II (Pilot Randomized Clinical Control Trial, 18 months)**: During Phase II we will be conducting a 18-month, pilot randomized clinical trial (RCT) of the adapted intervention (Phase I) to evaluate participant recruitment and retention rates, treatment and intervention satisfaction and estimate intervention effect sizes on our primary outcomes, A1C and self-management behaviors, as well as on secondary outcomes, diabetes social support and diabetes-related distress). This data will be collected baseline, completion of DSME (3 months), 9 months (approximate treatment termination), and 15 months (six month follow-up), see Table 1 for more details. Physiolocial testing will be done at St. Patrick Senior Center or Ypsilanti Seventh Day Adventist Church and information from self-report questionnaires will be collected telephonically, virtually, or at the senior center. All participants will be invited to the assessment battery, even if they did not complete the intervention. Participants will be randomized to either the adapted Peer Leader DSMES or a control group, all group sessoins will be held virtually over Zoom. In the PLDSMS arm, participants will receive 10 hours of diabetes self-management education (DSME) led by a Certified Diabetes Care and Education Specialist (CDCES), followed by 6 months of monthly 90-minute DSMS groups sessions led by two Peer Leaders (PL), with oversight from the CDCES. After the 6 monthly sessions of DSMS, participants and PLs will transition into a six-month period of ongoing support and be encouraged to foster ongoing DSMS through programs and initiatives that are meaningful to them. All PLDSMS intervention group sessions will be delivered in a group format with two groups of 15 participants each with each group assigned two PLs. Participants randomized to the control group will receive 10 hours of DSME led by a CDCES over 3 months. These participants will not receive any DSMS or ongoing support from the PLs. This was chosen as the control in order to 1) ensure that nay intervention effects are not due to provision of diabetes education alone, 2) minimize ethical concerns regarding assignment of underserved populations to receive a no-treatment control and 3) control for improvements due to attention and positive regard and expectancies for improvement due to participation in treatment.

**Phase III (Finalize for R01):** During Phase III will be comprised of three parts; conducting post-intervention interviews and focus groups with participants and stakeholders, statistical analysis, and dissemination of findings. Dissemination activities will include presentation of findings to academic and community forums. Within the academic arena, findings will be presented to the annual meeting of the American Diabetes Association in addition to the preparation of at minimum 3 manuscripts. Community-based dissemination of findings will include a report and presentation that will be shared electronically with researchers, community-supported, translational health research focused on recruiting and retaining African American men in diabetes research. Based on participant feedback and focus groups, a toolkit will be provided to St. Patrick Senior Center and will contain community resources, data collection tools to allow for continuation of the program, and functions staff need to play to sustain improvements in outcomes.

**Table 1 Study Timeline**


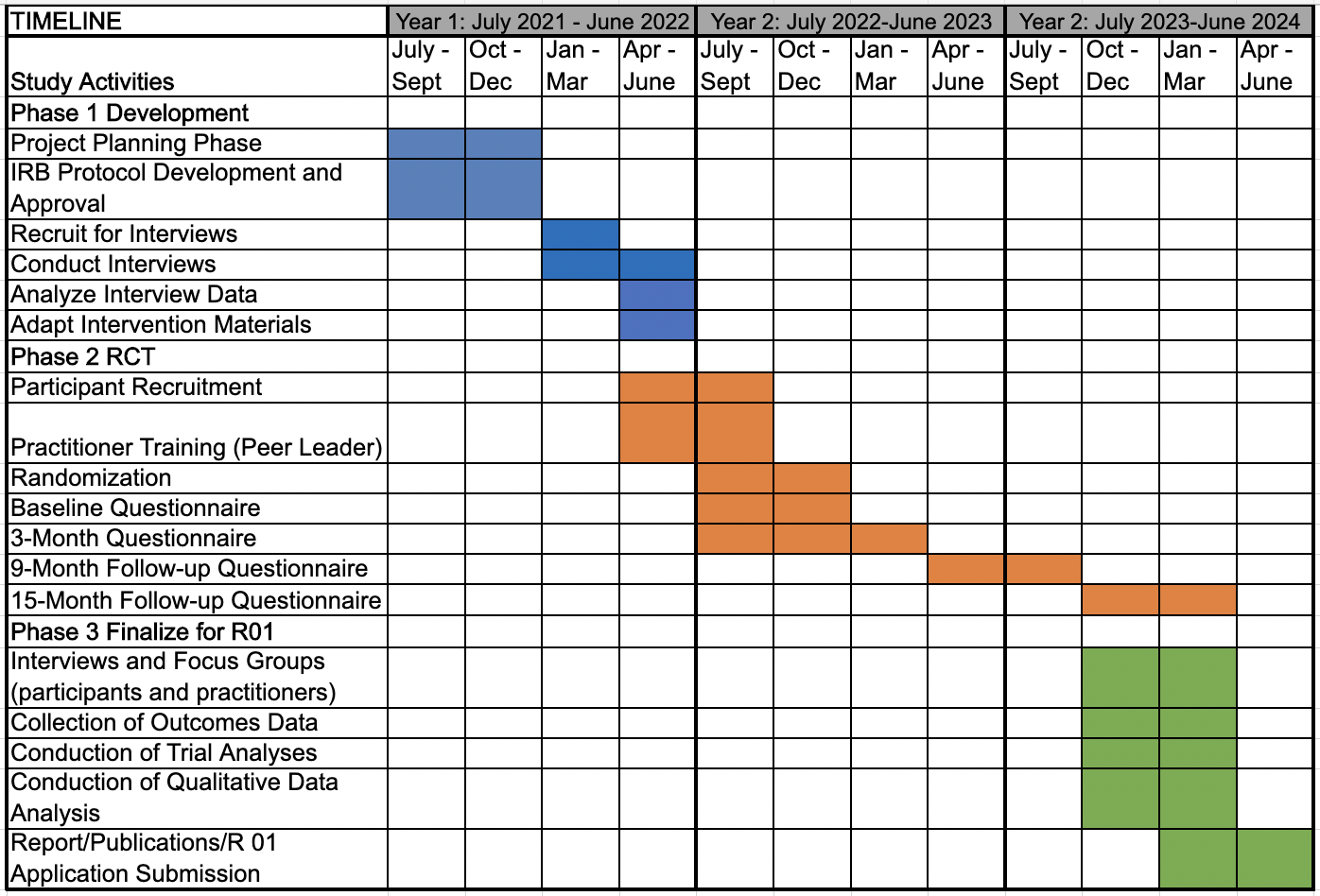


**2.0 BACKGROUND**

*“Well, the way I was raised men are strong and we don’t need to go to the doctor—we can take care of ourselves. When I first got diagnosed with diabetes, I was still trying to do that on my own without listening to everybody else.”* Black men are approximately twice as likely to have type 2 diabetes (diabetes) and to die from diabetes complications as compared to non-Hispanic White men.^1,2^ Yet, the statement above, from a Black male research participant, illustrates a unique and critical barrier encountered by Black men when managing their diabetes –the effect of gender role norms on diabetes self-management.

An emerging body of work demonstrates the critical role of gender in diabetes self-management.^3,-5^ For instance, our prior work shows that male gender norms and cultural expectations among Black individuals for male self-reliance creates barriers to asking for help from family members to support health behavior change, or following health advice given by medical professionals.^4,5^ For example, Black men with diabetes reported waiting until diabetes complications became severe (e.g., blurred vision, numbness in extremities) before seeking medical attention.^5^ These results are consistent with the broader literature in showing that the need to maintain a strong image to the outside world, and to maintain control of one’s own health served as barriers to engaging in healthy behaviors.^5^ Our work and that of others suggest that tailoring diabetes self-management education and support interventions to address the needs of Black men is critical to helping them to lead healthier lives.^4,5^

The National Standards for Diabetes Care [^6^](#_ENREF_6) and the National Standards for Diabetes Self-Management Education (DSME) and Support [^7^](#_ENREF_7) emphasize the importance of providing initial DSME **and** on-going diabetes self-management support (DSMS) to assist people with diabetes in maintaining effective self-management. DSMS interventions, including those delivered by professionals and non-professionals (e.g. peer leaders), improved A1C and self-management outcomes compared to control groups.^8-17^ Moreover, emerging research suggests that Black men with diabetes prefer peer-led, male-to-male interventions.^18-20^ Use of male interventionists to promote health behavior change in men is also consistent with approaches used for other chronic illnesses, such as HIV.^21^ Despite this fact, the majority of peer leaders and participants in DSMS studies are women,^22^ which creates a critical gap in our understanding of the utility of DSMS among men.^20,22^

Based on our previous work, the ***long-term goal*** of our research is to determine the most effective, practical, and sustainable approaches to provide ongoing DSMS that address the unique needs of Black men. The Praise study was a 15-month cluster-randomized controlled trial that examined the effects of peer-led DSMS on A1C and diabetes distress (100% Black, 25% male). Considering the success of Praise and other peer-led DSMS interventions,^8^ an opportunity exists to further tailor interventions to meet the needs of Black men in order to effectively reach, retain, and improve diabetes outcomes in this population. Therefore, the ***objective*** of this study is to adapt and evaluate the preliminary efficacy of a peer-led DSMS (PLDSMS) intervention for Black men with diabetes. To accomplish this objective, we will tailor an existing peer-led DSMS intervention, Praise,^23^ by 1) using male peer leaders as interventionists and 2) modifying the intervention content to focus on messaging appropriate for Black men. The proposed study includes a developmental phase (adaptation of the intervention with stakeholder feedback, followed by feasibility testing with Black men) and a validation phase [pilot randomized controlled trial (RCT)]. The RCT will be conducted with N=60 Black male adult residents of Detroit, MI. Men will be randomized to an enhanced usual care group or to the tailored PLDSMS. We ***hypothesize*** that 1) participants in the adapted PLDSMS approach will have improved outcomes over enhanced usual care (EUC), and 2) an evaluation of measures will confirm efficacy of the intervention. Our specific aims are:

**3.0 AIMS**

**Aim 1 (Phase I; HUM00200496): Intervention adaptation.** Adapt the Praise intervention for Black men with diabetes. Adaptation and refinement will involve conducting interviews to understand the structural, contextual and cultural factors impacting the implementation of a peer-led diabetes education and support intervention adapted for Black men with type 2 diabetes.

**Aim 2 (Phase II):** **Pilot RCT.** Conduct a pilot RCT of the adapted intervention to evaluate participant recruitment and retention rates, treatment and intervention satisfaction and estimate intervention effect sizes on our primary outcomes (i.e., A1C and self-management behaviors) as well as on secondary outcomes (i.e., diabetes social support and diabetes-related distress).

**Overall Impact:** Data from the pilot RCT will help refine recruitment strategies, training materials, and the implementation protocol to be used in a larger cluster RCT. Our study will also identify strategies to increase Black men’s participation in intervention research and improve dropout rates. This goal is in line with the mission of NIDDK to disseminate science-based information on diabetes, to improve people’s health and quality of life.

**4.0 PARTICIPANTS AND RECRUITMENT**

*Participants will be 64 Black/African American men ages 21 years or older and meet the inclusion criteria listed below. During the Phase II pilot we will individually randomize each participant with a 50/50 randomization scheme to either the peer led DSMS or a control group. Four of the participants will attend 30- hours of training and function as peer leaders during the intervention DSMES.*

**Inclusion Criteria for Peer Leaders:** Inclusion criteria for Peer Leaders includes Black/African American males, age 21 years or older with diagnosis of T2D for one year or longer with an 8^th^ grade education and transportation to attend training and program activities, willing to commit to 30 hours of training, willing to attend group delivered sessions, actively working on his own self-management goals, under the care of a physician for diabetes, and willing to serve as a peer leader.

**Inclusion Criteria for Participants:** Inclusion criteria for participants includes Black/African American males, age 21 years or older with diagnosis of T2D for six months, transportation to attend program activities, under the care of a physician for diabetes, and willing to attend group delivered sessions.

**Exclusion criteria**: We considered restricting eligibility to a higher-risk population of participants with A1c ≥ 8%. Preliminary data suggest that over 50% of the proposed study sample will have an A1c ≥ 8%. Focusing on all older Black men with T2D allows us to cast a wide net for secondary prevention and public health impact. Persons who meet eligibility criteria will be invited to participate in the baseline screening assessment. While we have chosen the above eligibility criteria based on previous work, we will make adjustments to the future, larger trial, based on results and feedback from our proposed pilot.

**Participant Recruitment**: We will put up flyers describing the study. The flyers will be posted at a Detroit-based senior center (St. Patrick’s), Ypsilanti Seventh Day Adventist, and Michigan Center for African American Aging Research Participant Resource Pool (MCUAAAR PRP) community-partners. The Healthier Black Elder Center will include a recruitment posting in their newsletter and email listings. Men interested in participating in the study will call a central office phone number or be identified on follow-up calls then they will be scheduled for screening. The MCUAAAR PRP is a research volunteer registry can be accessed by scholars conducting research of Black males, 55 years of age and older who meet their study criteria. Men with T2DM identified with the help of the MCUAAAR PRP will be called by study staff and invited to participate in the intervention. Additionally, we will have Dr. Linda Nyquist, run a search in the MICHR DataDirect PHI system to identify potential participants that meet study eligibility criteria. She will provide the team with the names, addresses, phone numbers, an email of potential participants on a password protected document. Potential participants will cold called and/or sent a recruitment letter via email. Study staff will provide follow up phone calls after the letters are sent to ensure potential participants received the email. Men interested in participating in the study will call a central office phone number or be identified on follow-up calls then they will be scheduled for the baseline health assessment. We also plan to use a snowball sampling approach in three ways. First, we will provide Phase I participants with a recruitment flyer. Second, we will provide a recruitment flyer to participants from previous study (HUM00190932) that expressed interest in being part of another peer leader DSMES study for Black men with type 2 diabetes. Furthermore, we will provide a recruitment flyer to other researchers. While we cannot guarantee the location of where the flyers will end up, we expect the flyer to be shared among personal and professional networks.

**Peer Leader Participant Recruitment:** Peer leaders will be recruited from the Phase 1 (HUM00200496) interviews, participants from a previous study (HUM00190932) that expressed interest in being a peer leader or identified by St. Patrick Center staff.

**Potential problems:** One issue may be attrition. It is important to note that our team has high recruitment and retention rates among Black men in our target recruitment region. Dr. Hawkins previously conducted studies using Black men with diabetes recruited from the PRP, recruiting and retaining 30 out of 32 men for diabetes focused qualitative research. Our research team has substantial experience with retention of high-risk samples that are similar in nature to the proposed sample. To ensure retention: 1) Data collection sessions will be completed at the community center in order to maximize the convenience of data collection for subjects and 2) multiple techniques will be used to increase the likelihood that participants will keep their data collection appointments, including advanced scheduling, reminder letters and phone reminders.

**5.0 PROCEDURES**

**Screening** Individuals that are interested in participating in the study will be asked to complete a phone screen with a study team member. During the phone screen, the research staff will read a brief recruitment script and ask if the participant is interested in participating. If the individual is interested and willing, they are asked to complete a phone screen. The phone screen can be scheduled for immediately following the recruitment script or at a time convenient for the potential participant.

The phone screen will assess the following: basic demographic information and self-identified T2D diagnosis. Individuals will be eligible for a baseline interview if they meet demographic criteria (male, self-identified as African American/black, and 21 years or older), self-report having Type 2 Diabetes.

Upon completion of the phone screen, the study team member will inform the individual if they are eligible to complete the baseline interview. If the individual is eligible, a baseline assessment will be scheduled, and details will be given to the individual. The purpose of this initial screen is to invite research candidates to additional eligibility screening (Baseline Assessment) having met initial criteria and for the research candidates to provide consent to answering questions at the in-person Baseline Assessment which involve Private Health Information, to be used for determining eligibility.

**Baseline Assessment** (Assessment Battery) All individuals that meet initial phone screening criteria and agree to participate will complete a baseline assessment. Prior to completing the baseline assessment, participants will read through and sign the informed consent either in person or virtually. Participants will be able to complete this process in person at St. Patrick Senior Center or Ypsilanti Seventh Day Adventist Church or by using SignNow. Study team members will be available to answer questions about the informed consent either in person or over the phone. The physiological testing portion of the baseline assessment will be held in person at either St. Patrick Senior Center or Ypsilanti Seventh Day Adventist Church and the self-report questionnaire will be completed by the participant virtually or over the phone with a study team member. Baseline assessments will last about two hours total. The PI will train all research staff in standardized data collection. Trained research staff will take biometric data of participants including height, weight, blood pressure, and A1c. Participant A1c values will be analyzed on site using a DCA Vantage Analyzer. All participants, including peer leader participants, will be paid $50 for completing the assessment and the same assessments at T0 (baseline), T1 (completion of DSME), T2 (treatment termination), and T3 (six-month follow-up).

**Randomization** Participants will be randomized to the peer leader diabetes self-management support (DSMS) group or a control group using a 50/50 randomization scheme. Participants will be aware if they are in the intervention group or the control group.

**Intervention (Peer Leader DSMS n=30)** Participants randomized to the peer led DSMS group will receive 10 hours of diabetes self-management education (DSME) delivered by a certified diabetes care and education specialists (CDCES) and co-facilitated by a peer leader (PL). DSME will take place over three months and will be delivered via the Zoom for Health a U-M service may be used for Protected Health Information (PHI, regulated by HIPPA). Next, participants will transition into 6 90-minute monthly PL led DSMS sessions intentionally designed for older Black men with T2D. The CDCES will provide oversight to the PL and be available by phone if any clinical questions arise during the session. Following DSMS, participants and PLs will transition into a 6-month period of ongoing support. To ensure treatment fidelity, three DSMS sessions will be selected at random and recorded and rated for fidelity by our research team.

**Control Group (Control n=30)** Participants randomized to the control group will receive 10 hours of group delivered DSME via the Zoom for Health U-M service; however, they will not receive any DSMS or ongoing support from PLs and the PL will not participate in the DSME sessions. Based on several years of experience in Detroit, providing all participants with DSME and educational materials minimizes ethical concerns regarding assignment of underserved populations to receive a no-treatment control.

**Peer Leaders (n=4)** Participants recruited to be PLs for the intervention group will attend PL training (described below), co-facilitate DSME sessions with a CDCES, lead DSMS sessions and ongoing support, and complete the same battery of assessments as all other participants in the study.

**Peer Leader Training:** The peer leader training curriculum will be based on materials used by Dr. Piatt and her research group. Thirty hours of training will be conducted over 3 months. PLs will receive training in facilitation skills, coping strategies, and empowerment-based communication skills. As noted in Phase 1, training will be adapted to include a focus on men’s health issues and updated/revised by CDCES’s to ensure content covered adequately prepares the Peer Leaders with the skills needed to facilitate DSMES groups. Training will be group-based and include both the knowledge and skills needed to implement empowerment-based DSMS. A CDCES who was involved in the development and implementation of the training curriculum in previous projects, will conduct the training. This training will support the PLs in developing communication, facilitation, and behavior change skills and opportunities to apply those skills in an experimental setting. To ensure that PLs are supported, quarterly meetings will be held with all PLs so that they may exchange information and support each other. As noted above, it is expected that this content will be adapted to include men’s health concerns based on Phase 1. PLs will be identified from interviews and based on recommendations from the senior center. PLs will be compensated at $10/ hour for the training, DSME, and DSMS to defray the cost of their time and expenses. PLs will not be compensated for ongoing support to mimic the real-world scenario of when the research grant is over.

**Follow-Ups** Follow up assessments for both the peer led DSMS group and control group will occur at 3 months (completion of DSME, T1), 9 months (approximate treatment termination, T2), and 15 months (six month follow up, T3). All participants, including peer leader participants, will return to the St. Patrick’s Senior Center for physiological testing and complete the self-report questionnaire telephonically, or virtually. The same measurements and surveys will be completed. Participants will be compensated $50 for completing each assessment. Data will be stored in REDCap, a secure, web-based application hosted at UM, and a Microsoft Access database.

**Maintenance of Samples** Strategies will be used to encourage attendance of group sessions and to complete follow up interviews. Reminder emails, texts and/or calls will be sent before each session to remind participants by the study team. In addition, the study team will collect participant contact information from baseline to follow up phase. The study team will also ask participants to provide additional contact person(s) that the study team can contact if they cannot reach the participant.

**Participant Retention** The following procedures will be used to minimize participant attrition: 1) Data collection sessions will be completed at the Senior Center site in order to maximize the convenience of data collection for subjects, and 2) multiple techniques are used to increase the likelihood that participants will keep their data collection appointments, including advanced scheduling, multiple reminder letters, and phone reminders. Participants who withdraw will still be asked to participate in study data.

**Data Collection & Data Safety** for Phase I (HUM00200496) and III (Development and Finalization), data will be collected through transcriptions of the audio recorded interviews. For Phase II (intervention), data will be collected through the following methods: finger stick capillary blood samples, blood pressure measurements, weight, height, self-reported surveys, audio recorded treatment sessions, and transcribed treatment session recordings. Data that are obtained specifically for research purposes will be collected only with informed consent. All data will be collected over the course of the award period.

At the time of study enrollment, participants will be assigned a study identification number to be used in all study materials and data for the duration of the study. All identifying information will be separated from the data and laboratory values. A master list that contains participants names and study identification number will be kept in a locked filing cabinet within a locked office in the School of Social Work at the University of Michigan. Audiotapes of treatment sessions will be stored securely on a password protected computer only accessible to study personnel and will be destroyed upon study completion. We will use DropBox, a secure platform to share these recordings, transcriptions, and other study data between study team members.

The principal investigator (Dr. Hawkins) and the research assistants will be the only persons who have access to the file linking study participant identification number to each subject, and this will be stored separately from study data. Participants will be assured that all data they provide to the study will be confidential to this study, unless it is necessary to “alert” the patient and possibly also their physician because of a laboratory value outside of the normal ranges that reflects a risk requiring immediate medical attention. All reports will use aggregate data. Subject names or other identifiers will not be reported. All quotes shared collected from the interviews will be de-identified for privacy. No persons from the recruitment sites will handle or have access to personal health information or participant survey data.

Data will be stored in REDCap, a secure, web-based application hosted at the University of Michigan as well as a Microsoft Access database. Analyses will be conducted using Atlas.ti. This data will be used to conduct a final refinement of treatment content as needed.

To ensure the proper monitoring of the safety of all participants and the quality of data collected, a Data Safety and Monitoring Board (DSMB) will be established that will follow techniques suggested in the literature (Damocles, et al, 2005). The board will consist of Drs. Hawkins, Piatt, and Herman. In addition, five outside members, who are not involved with the study, will serve on the DSMB as voting members and will be identified at a later date. Voting members will consist of University professors with experience and expertise in clinical diabetes intervention research to ensure consistency and quality of input.

**6.0 GROUP LEADERS & TRAINING**

To ensure staff participation in data safety and monitoring activities, all members of the project team (research assistants and staff) will be trained on the specifics of the data safety and monitoring plan. Field staff will be trained on what constitutes an adverse event to a participant and instructed to report any adverse events immediately to the principal investigator, Dr. Jaclynn Hawkins.

All questionnaires and instrumentations are standardized measures that have been used in our own trials and in other diabetes research and there are no significant risks anticipated related to the completion of them. However, breaks will be given as needed to reduce fatigue, or measures read to adolescents, and research assistants will be appropriately trained to obtain personal information in a sensitive fashion. Research staff will be trained in research ethics, confidentiality protection, and HIPAA prior to and throughout the study period. All CDCESs in this study are either a Registered Dietitian Nutritionist RDN) or Registered Nurse (RN) and are certified through the Certification Board for Diabetes Care and Education. The extensive CDCES certification process ensures health care professionals possess comprehensive knowledge and experience in prediabetes, diabetes prevention, and diabetes management. Katherine Kloss, RDN, CDCES has been working with people with diabetes for 6 years and a CDCES for 2 years. Robin Nwankwo, MPH, RDN, CDCES has been working with people with diabetes for 28 years and a CDCES for 24 years.

The PI will monitor adverse events throughout the clinical trial period. To ensure staff participation in data safety and monitoring activities, all members of the project team (research assistants and staff) will be trained on the specifics of the data safety and monitoring plan. Field staff will be trained on what constitutes an adverse event to a participant and instructed to report any adverse events immediately to the principal investigator, Dr. Jaclynn Hawkins.

The principal and co-investigators on the proposed study make up a trans-disciplinary, accomplished and collaborative team of community-based behavioral and clinical researchers. Together, our research team has a strong history of successfully implementing and publishing our work regarding diabetes interventions in high-risk communities. Most notably, Drs. Piatt and Herman of the Michigan Center for Diabetes Translational Research (MCDTR), collaborated on Praise I and II, T2D randomized controlled trials of the effectiveness of church-based diabetes self-management support being conducted with Black adults in Toledo, Flint, and Metro Detroit of which Dr. Piatt is PI. Dr. Hawkins is currently a co-investigator on Praise 2 (R01DK104733-02). Also, as part of the MCDTR, Dr. Hawkins is an early career trainee and Dr. Piatt served as Dr. Hawkins’ primary mentor for the last 3 years. All are members of BRIDGE.

**7.0 MEASURES**

Primary and secondary outcome measures will be measured and collected during Phase II (Intervention – Pilot RCT)

**Primary Outcome Measures:**

Glycemic control will be measured using hemoglobin A1c (A1c) and collected using the DCA 2000 point-of-care testing instrument in order to maintain consistency of measurement over time.

Regimen adherence will be measured using the Perceived Diabetes Self-Management Scale, s self-report questionnaire used to measure a broad range of management behaviors, such as insulin management, dietary management, blood glucose monitoring, and symptom response.

**Secondary Outcome Measures**

Depression, Body Mass Index (BMI), blood pressure, Diabetes Social Support, Diabetes-related Distress, the SF-12, Diabetes Quality of Life and adherence to gender norms. Other variables include sociodemographic, behavioral, psychosocial, and health services utilization measures (Table 2).

| **Table 2. Primary and Secondary Outcome Measures** | | |
| --- | --- | --- |
| ***Anthropometric Data and Clinical Data*** | | |
| **Measure** | **When Collected** | **Source of Measure** |
| Hemoglobin A1c | Baseline, 3, 9, 15 | Primary data collection |
| Height (inches) and weight (lbs) | Baseline, 3, 9, 15 | Primary data collection |
| BMI (kg/m2) A1c (%), BP (mmHg) | Baseline, 3, 9, 15 | Primary data collection |
| Medication use | Baseline, 3, 9, 15 | Primary data collection |
| ***Survey Data*** |  |  |
| **Measure** | **When Collected** | **Source of Measure** |
| Sociodemographic characteristics, healthcare utilization, comorbidities | Baseline, 3, 9, 15 | Diabetes Care Profile |
| Diabetes quality of life, General quality of lie | Baseline, 3, 9, 15 | Type 2 Diabetes Distress Assessment System, SF-12 |
| Depressive symptom severity | Baseline, 3, 9, 15 | Patient Health Questionnaire (PHQ-9) |
| Empowerment | Baseline, 3, 9, 15 | Diabetes Empowerment Scale Short Form |
| Perceived social support | Baseline, 3, 9, 15 | Social Support Questionnaire |
| Self-care barriers and resources | Baseline, 3, 9, 15 | Chronic Illness Resources Survey |
| Self-Care behaviors | Baseline, 3, 9, 15 | Perceived Diabetes Self-Management |
| Masculinity norms scale | Baseline, 3, 9, 15 | Conformity to Masculine Inventory (CMNI-30) |

**Specific Aim 1**: Qualitative analyses will be conducted during Phase I and in the post-intervention interviews during Phase III. Interviews will be recorded and transcribed. We will develop codes utilizing a grounded theory approach and will start with the formulation of categories and definitions developed directly from the text. Through this process a coding a manual and definitions will be finalized. The refined manual will be used to guide ongoing coding and pairs of coders will read subsequent transcripts keeping codes that achieve 80% agreement on code application. Analyses will be conducted using Atlas.ti. For Phase I (development), these data will be used to conduct a final refinement of treatment content as needed. For Phase III (post intervention), intervention components from the perspective of participants will be assessed using interviews to establish comprehension, acceptability and feasibility of the intervention and to detect any domain-specific issues.

**Specific Aim 2:** We will recruit a sample of 64 Black men, 30 in the intervention arm, 30 in the control arm and 4 Peer Leaders. The effect of the intervention on diabetes management and A1C (primary outcomes) will be assessed using mixed-effects models. Diabetes management and A1C at baseline and the three follow-ups will be used as the dependent variables. Independent variables include intervention group, time of assessment, and interaction between time and intervention group. To take into account correlation between observations, random intercepts and slopes will be included into the model. In the analysis we will follow intention-to-treat principles. Analyses will be conducted with adjusting for stratifying variables (i.e., age group).

**Power Analysis:** Assuming 20% attrition, we expect a final sample size of 48, approximately 12 per group (with 2 groups in the treatment arm and 2 groups in the control arm). If we assume correlations of 0.25 between successive measurements of HbA1c, then this sample size will yield power of 0.8 to detect a difference of 0.6 standard deviation between average values of HbA1c in treatment and control groups.

**10.0 PROTECTION OF HUMAN PARTICIPANTS**

The protocol for this study will meet approval by the University of Michigan Institutional Review Board prior to initiating any of the described study activities.

**Human Participants Involvement and Characteristics Removing Participants from the Protocol:** Respondents who meet eligibility criteria will be invited to participate in the baseline screening assessment. While we have chosen the above eligibility criteria based on previous work, we will make adjustments to the future, larger trial, based on the results and feedback from our proposed pilot.

**Sources of Materials**: For Phase I (development) and Phase III (finalize for R01), data will be collected through focus groups and interviews and transcriptions of these focus groups and interviews. For Phase II (intervention), data will be collected through the following methods: self-report surveys, finger stick capillary blood samples, blood pressure measurements, weight, and height. Data that are obtained specifically for research purposes during all three phases will be collected only with informed consent. All data will be collected over the course of the award period.

**Potential Risks** Participation in this study involves minimal foreseeable risks. Participants will be asked to provide finger stick capillary blood samples during assessments. Risks associated with finger stick capillary blood draws include: minor discomfort from obtaining the blood sample, minor pain, bruising, or bleeding at the puncture site similar to any other routine blood sample collections. With self-report surveys, there is also the small risk that prompting patients to review their diabetes care practices and providing them with feedback about their diabetes-related health outcomes (e.g., A1C, blood pressure) could cause some emotional discomfort or anxiety. Such discomfort would likely prime patients and their primary care physician or group facilitator to address any problems identified. Other risks include breach of confidentiality of study data.

**Protection Against Risk**

All questionnaires and instrumentations are standardized measures that have been used in our own trials and in other diabetes research and there are no significant risks anticipated related to the completion of them. However, breaks will be given as needed to reduce fatigue, or measures read to adolescents, and research assistants will be appropriately trained to obtain personal information in a sensitive fashion. Research staff will be trained in research ethics, confidentiality protection, and HIPAA prior to and throughout the study period. All peer leaders must pass standardized training prior to providing service to participants and will also be trained in protection of participant confidentiality.

At the time of study enrollment, participants will be assigned a study identification number to be used in all study materials and data for the duration of the study. All identifying information will be separated from the data and laboratory values. A master list that contains participants names and study identification number will be kept in a locked filing cabinet in the School of Social Work. Audio/visual recordings of the intervention for fidelity purposes will also be stored securely on a password protected computer only accessible to study personnel and will be destroyed upon study completion. The principal investigator (Dr. Hawkins) and the research assistant will be the only persons who have access to the file linking study ID# to each subject. Participants will be assured that all data they provide to the study will be confidential, unless it is necessary to “alert” both the patient and their physician because of a laboratory value outside of the normal ranges that reflects a risk requiring immediate attention. Participant’s physicians will receive a report of participant laboratory values after each assessment. All reports will use aggregate data. Subject names or other identifiers will not be reported. No persons from the senior center or other community-based location will handle or have access to personal health information or participant survey data.

**Potential Benefits of The Proposed Research to The Participant and Others**: Participants will have the potential to benefit from the study by receiving free diabetes self-management education and support from the intervention and also an opportunity to discuss barriers and facilitators to recruitment and retention of men and women in a large-scale intervention. There are also benefits to society from the research through its potential to improve diabetes self-management interventions for persons with Type 2 diabetes. We feel the benefits of participating in this study significantly outweigh the risks.

**11.0 DATA AND SAFETY MONITORING PLAN**

**Adverse Events:** The PI will monitor adverse events throughout the clinical trial period. To ensure staff participation in data safety and monitoring activities, all members of the project team (research assistants and staff) will be trained on the specifics of the data safety and monitoring plan. Field staff will be trained on what constitutes an adverse event to a participant and instructed to report any adverse events immediately to the principal investigator, Dr. Jaclynn Hawkins.

For the purposes of this study, adverse events will be considered any undesirable sign, symptom, or medical condition occurring during the study, whether or not related to the intervention. Adverse events include new events not present during the training period or events that were present during the training period but increased in severity over time. Each adverse event will be recorded and assessed for its date of onset, duration, severity, seriousness, and relationship to study treatment, and any action/treatment that is required. All adverse events will be collected, analyzed, and monitored using an adverse event form. Furthermore, the committee will establish “alert” values for A1C and blood pressure. The PI will notify the subject and the subject’s physician whenever there are laboratory results above these values because of the clinical implications of a value substantially out of normal range.

All serious medical events will be reported within one business day of their identification. All serious, fatal or life-threatening adverse events will be reported to UM IRB and the NIH within 24 hours of its identification. All causes of death are considered to be serious medical events. Unexpected moderate or severe adverse events will be reported in writing to the IRB and NIH. Serious medical events will be collected throughout the intervention phase of the study. The PI, along with the study physician, Bill Herman, and/or co-Investigator Gretchen Piatt, will adjudicate whether or not each serious adverse event may be attributable to study participation. Events that involve an unexpected adverse event and are possibly or probably related to participation in the study will be reported to the IRB at University of Michigan within one business day. Annual reporting of aggregate adverse events to the IRB and NIH will be performed. All research project personnel will complete training in the protection of human research participants. The PI and research assistant will verify appropriate reporting of adverse events, quality of data collection, and adherence to the study protocol. Participant dropout rate will also be monitored and reviewed for needs or trends based on specific participant characteristics.

**Data Safety & Monitoring Board:** The DSMB will hold a minimum of three conference calls over the award period (approximately two hours per call) to discuss the progress of the intervention and review research results, if applicable. At the first meeting, the board will elect a chair of the DSMB. To facilitate these conference calls, the principal investigator will prepare a report on the progress of the project to date. This report will be circulated well in advance of the conference call to allow all members ample time to read it. These calls will be scheduled and organized by the study coordinator and will be held at a time convenient for all members.

The DSMB is responsible for assuring that study participants are not exposed to unnecessary or unreasonable risks and that the study is being conducted according to high scientific and ethical standards. Specifically, the DSMB will:

1. Assess the performance of the study with respect to subject recruitment, retention and follow-up, protocol adherence, and data quality and completeness, in order to ensure the likelihood of successful and timely milestone completion.

2. Monitor interim data regarding the safety of the study, including adverse events. The DSMB may, at its discretion, examine effectiveness data as well.

3. Review abstract and publications of main findings prior to submission to ensure the study is being reported appropriately.

4. Review and consider any protocol modifications or ancillary studies proposed by the study investigators after the main study begins to ensure that these do not negatively impact on the main trial.

5. Advise the NIA and the study investigators as to whether a protocol should continue as scheduled or undergo a modification due to a finding from the monitoring process.

6. Make recommendations to the NIDDK and principal investigator concerning continuation or conclusion of the trial.

**12.0 STUDY SITES**

**Empowerment-based DSME/S sessions:** All DSME/S sessions will be held virtually via Zoom for Health. Baseline, post-treatment and follow-up assessment self-report questionnaire will take place telephonically or virtually and the physiological part of those assessments will take place at St. Patrick’s Senior Center, a non-profit that provides comprehensive services to a diverse population of more than 2,000 seniors living throughout metropolitan Detroit or Ypsilanti Seventh Day Adventist Church.

**Elements Unique to this Site** In each location, rooms with doors and telephones will be available to facilitate privacy, confidentiality, and safety of assessments.
