# Supplementary material for: Michigan men’s diabetes project II: Protocol for peer-led diabetes self-management education and long-term support in Black men
Source: PLoS One. 2023 Mar 2;18(3):e0277733. doi: 10.1371/journal.pone.0277733 (PMC9980828; doi:10.1371/journal.pone.0277733)
Supplement: Peer review history

# Editor report: Decision Letter 0

**DOI:** 10.1371/journal.pone.0277733.r001

**Authors:** Alexandra Schaefer

10 Feb 2023

Michigan men’s diabetes project II: protocol for peer-led diabetes self-management education and long-term support in Black men

PONE-D-22-29589

Dear Dr. Hawkins,

We’re pleased to inform you that your manuscript has been judged scientifically suitable for publication and will be formally accepted for publication once it meets all outstanding technical requirements.

Within one week, you’ll receive an e-mail detailing the required amendments. When these have been addressed, you’ll receive a formal acceptance letter and your manuscript will be scheduled for publication.

An invoice for payment will follow shortly after the formal acceptance. To ensure an efficient process, please log into Editorial Manager at http://www.editorialmanager.com/pone/, click the 'Update My Information' link at the top of the page, and double check that your user information is up-to-date. If you have any billing related questions, please contact our Author Billing department directly at authorbilling@plos.org.

If your institution or institutions have a press office, please notify them about your upcoming paper to help maximize its impact. If they’ll be preparing press materials, please inform our press team as soon as possible -- no later than 48 hours after receiving the formal acceptance. Your manuscript will remain under strict press embargo until 2 pm Eastern Time on the date of publication. For more information, please contact onepress@plos.org.

Kind regards,

Alex Schaefer, PhD

Associate Editor

PLOS ONE

Additional Editor Comments:

1) Please ensure to that on page 6, line 7 of the Objectives section reference 24 is added as follows: 

"Our previous study, Michigan Men’s Diabetes Project (MenD) [24], measured health outcomes for Black men with T2D who participated in peer-led support group interventions."

# Editor report: Acceptance letter

**DOI:** 10.1371/journal.pone.0277733.r002

**Authors:** Alexandra Schaefer

16 Feb 2023

PONE-D-22-29589 

Michigan men’s diabetes project II: protocol for peer-led diabetes self-management education and long-term support in Black men 

Dear Dr. Hawkins:

I'm pleased to inform you that your manuscript has been deemed suitable for publication in PLOS ONE. Congratulations! Your manuscript is now with our production department. 

If your institution or institutions have a press office, please let them know about your upcoming paper now to help maximize its impact. If they'll be preparing press materials, please inform our press team within the next 48 hours. Your manuscript will remain under strict press embargo until 2 pm Eastern Time on the date of publication. For more information please contact onepress@plos.org.

If we can help with anything else, please email us at plosone@plos.org. 

Thank you for submitting your work to PLOS ONE and supporting open access. 

Kind regards, 

PLOS ONE Editorial Office Staff

on behalf of

Alexandra Schaefer 

Staff Editor

PLOS ONE
